# Functional data analysis of lower-limb joint kinematics during badminton lunges under fatigue

**DOI:** 10.3389/fbioe.2026.1741767

**Published:** 2026-02-06

**Authors:** Yuling Fang, Xingchen Zhang, Yang Sun, Hanbing Wu, Huanran Pei, Jingwen Gao, Jiujiang Liu, Qing Zhu, Yuan Gao

**Affiliations:** School of Physical Education, Yanshan University, Qinhuangdao, China

**Keywords:** badminton, fatigue, functional data analysis, lower limb biomechanics, lunge

## Abstract

**Objective:**

This study applied Functional Data Analysis (FDA) to investigate the effects of fatigue on lower-limb joint kinematics during badminton lunges.

**Methods:**

Seventeen elite male badminton players executed both forward and 45° sideways forehand lunges before and after a fatigue protocol. Three-dimensional kinematic data of the lower limbs were collected using a motion capture system synchronized with force plates. Functional principal component analysis (FPCA) was employed to reduce dimensionality and extract key features from the time-series curves of hip, knee, and ankle joint angles. Paired-samples t-tests were used to compare the principal component scores before and after the induction of fatigue.

**Results:**

Fatigue significantly altered lower-limb movement patterns. Sagittal-plane analyses revealed a decreased range of motion in hip and knee flexion, contrasted with an increased range of ankle dorsiflexion (*p* < 0.05). In the coronal plane, hip and knee abduction-adduction ranges decreased, while ankle inversion-eversion ranges increased (*p* < 0.05).

**Conclusion:**

Fatigue triggered a “top-down” compensatory response: proximal joints stiffened to stabilize, while the ankle enhanced flexibility to compensate. This “proximal-stiffening, distal-mobility” pattern maintained performance but may have redistributed loads, potentially raising the risk of ACL and lateral ankle ligament injuries. These findings help guide fatigue-specific training and injury-prevention strategies.

## 1 Introduction

The continuous evolution of technique and tactics in badminton has progressively underscored the critical importance of footwork, establishing it as a pivotal factor determining competitive outcomes (Wei and Liu, 2008). The lunge, a fundamental and frequently utilized footwork movement, constitutes approximately 15% of technical actions in elite-level matches, with its prevalence increasing alongside competitive standards (Kuntze et al., 2010). In practice, the progression of matches coupled with physical exertion consistently induces significant neuromuscular fatigue in athletes (Chan, 2019). Fatigue not only diminishes movement velocity and reaction time (Kudzia et al., 2023) but also substantially compromises motor control, dynamic postural stability (Ritzmann et al., 2016), and joint proprioception (Rozzi et al., 1999; Hewett et al., 2005), thereby precipitating a failure in overall coordination mechanisms (Romanazzi et al., 2015). Of critical importance, fatigue induces functional laxity in the periarticular musculature and ligamentous systems, alters muscle force production patterns, and disrupts stiffness regulation, directly impairing functional joint stability (Butler et al., 2003; Ri et al., 2008). Wikstrom et al. (2004) noted that successful landing actions necessitate sufficient muscular strength, joint stability, and postural balance—inherent protective mechanisms to avoid injury. Consequently, fatigue not only degrades the quality of technical execution but also markedly elevates the risk of sports-related injuries.

Current research on the lunge has investigated various determinants, such as lower-limb strength (Cronin et al., 2003), athlete expertise (Mei et al., 2017), and footwear properties (Wei, 2009), forming a preliminary framework for understanding its performance. However, prevailing studies are largely founded on non-fatigued, idealized models and rely on discrete, cross-sectional parameter analyses, failing to capture the continuous dynamics of the movement within authentic competitive contexts. To systematically uncover the continuous evolution of the lunge under fatigue, this study introduces FDA. This methodology is extensively applied in sports injury diagnosis (Donoghue et al., 2008) and technique analysis (Dona et al., 2009). FDA models the observations for each sample as continuous function curves, rather than as traditional discrete data points (Ryan et al., 2006). Its core principle utilizes the function as the fundamental unit of analysis, emphasizing a holistic characterization of the intrinsic dynamic structure and temporal evolution of the data. This approach reveals sports biomechanical patterns elusive to conventional techniques by leveraging higher information completeness (Harrison et al., 2007). This analytical framework not only overcomes the dependency of traditional statistical methods on discrete features but also aligns more closely with the time-evolving nature and energy constraints inherent to the human movement system (Bai-fa et al., 2019). Given the objective of this study, namely to understand the continuous, time-varying adaptation mechanisms under fatigue, FDA provides a particularly suitable analytical framework.

Based on this foundation, the present study aims to employ Functional Data Analysis (FDA) to systematically elucidate the time-varying joint kinematics of badminton lunges under fatigue, thereby providing a theoretical basis and practical guidance for technical optimization and injury prevention in fatigued athletes. We hypothesize that fatigue will increase the complexity of lower-limb joint angle dynamics and that the central nervous system will demonstrate adaptive changes following fatigue.

## 2 Subjects and methods

### 2.1 Subjects

A total of 17 healthy, high-level male badminton players were recruited for this study (age: 22.25 ± 1.48 years; height: 180.33 ± 3.65 cm; body mass: 73.33 ± 5.99 kg; training experience: ≥6 years). The inclusion criteria were: (1) good health with normal motor function; (2) no engagement in strenuous exercise within 24 h preceding the tests and no subjective sense of muscle fatigue; (3) self-reported right-hand dominance and right leg dominance. The study protocol received approval from the Institutional Review Board of Qinhuangdao First People’s Hospital (Approval No.: 2025K-126-01), and written informed consent was obtained from all participants prior to their involvement.

### 2.2 Data collection

Kinematic and kinetic data were synchronously collected using a three-dimensional motion capture system (Qualisys, Sweden) integrated with four Kistler force plates. The motion capture system, comprising eight infrared high-speed cameras (Model A12), recorded movement data at 200 Hz, while the force plates acquired ground reaction force data at 2,000 Hz.

### 2.3 Testing protocol

**FIGURE 1:**
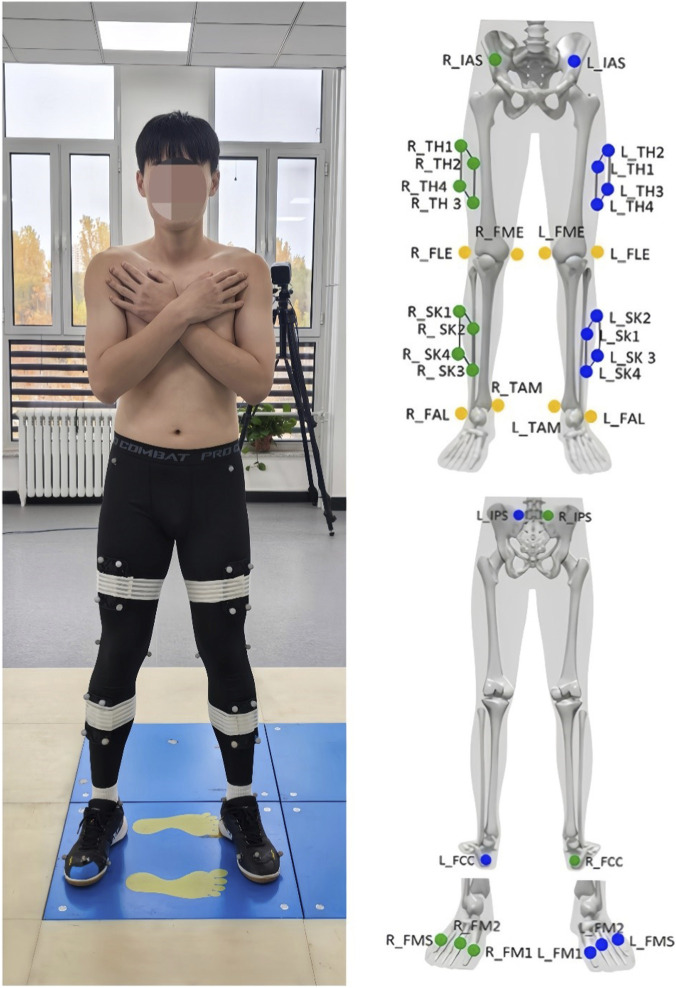
Anatomical landmarks of the lower extremity.

After being fully informed of the experimental procedures, participants changed into standardized tight-fitting clothing and professional badminton shoes provided by the laboratory. Following this, a single experimenter placed 36 passive reflective markers on key anatomical landmarks of each participant’s body surface, adhering to the specifications required for the Visual3D biomechanical modeling system, as illustrated in Figure 1. Subsequently, participants performed a brief warm-up and practiced the specified forehand lunge tasks to acclimate to the testing environment and requirements.

**FIGURE 2:**
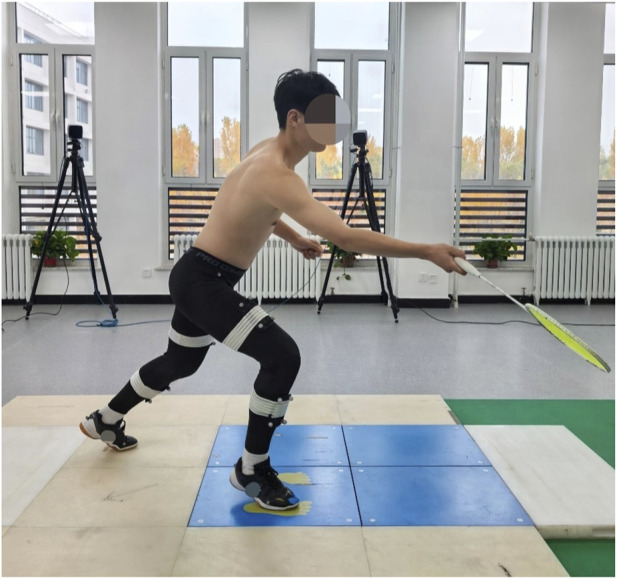
Forehand lunge.

The test movement was a right forward forehand lunge (Figure 2), conducted under two conditions: straight forward (0°) and 45° forehand diagonal directions. Upon the experimenter’s verbal “start” command, participants initiated the movement at a self-selected speed, performed a forward lunge, and simulated a net lift shot. The landing foot (i.e., the stepping foot) was required to contact the force plate completely within its boundaries without aiming precisely at the central area. Participants were instructed to step naturally and comfortably, with the sole constraint of avoiding contact on or beyond the edges of the force plate to ensure complete and valid ground reaction force data acquisition. The contralateral foot remained positioned on an extension plate adjacent to the force plate to simulate an authentic hitting posture. Three consecutive successful trials were collected for each movement direction. To ensure data quality and prevent cumulative fatigue, a rest period of no less than 5 min was provided after each successful trial collection.

### 2.4 Fatigue protocol

Following the initial pre-fatigue tests, participants immediately underwent a fatigue protocol designed to simulate the high-intensity intermittent activity of a competitive match. The protocol required participants to perform a set of 6 × 10-m shuttle runs, immediately followed by five consecutive maximal vertical jumps. This sequence was repeated until the following fatigue criteria were met: the objective criterion was an average height of five consecutive vertical jumps falling below 70% of their pre-test baseline maximum (Liu et al., 2023; Wang et al., 2020); the subjective criterion was a Rating of Perceived Exertion (RPE, 6–20 scale) reaching 17 or higher. Heart rate was monitored throughout the protocol for safety. The test was terminated immediately if a participant’s heart rate reached their age-predicted maximum (calculated as 220 - age).

### 2.5 Phase division

**FIGURE 3:**
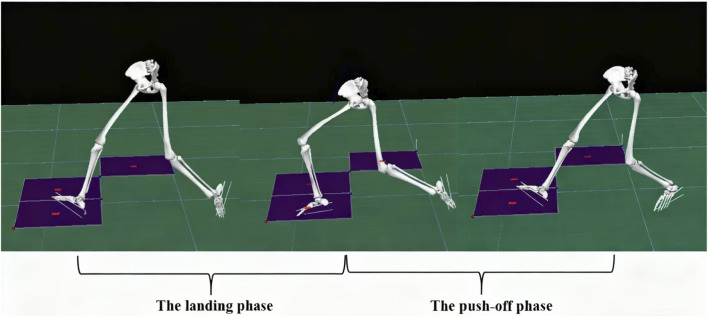
Diagram of the landing and push-off phase division.

As shown in Figure 3, the lunge movement was divided into two distinct phases: the landing phase and the push-off phase. The landing phase was defined as the interval from initial foot contact (vertical ground reaction force, GRF ≥10 N) to maximum knee flexion of the support leg. The push-off phase was defined as the period from maximum knee flexion until toe-off (GRF ≤10 N) of the support leg.

### 2.6 Data processing

A participant-specific lower limb model was developed according to the CAST (Calibrated Anatomical System Technique) lower limb model protocol. Coordinate systems for the hip, thigh, shank, and foot were defined using the infrared reflective markers, which were combined to construct the model. The extracted kinematic data were then imported into Visual 3D software and smoothed using a 4th-order low-pass Butterworth filter with a cutoff frequency of 16 Hz. Subsequently, the lower limb joint angles were time-normalized to 100% of the movement cycle duration. FDA was subsequently implemented in MATLAB using its dedicated FPCA toolkit. The joint angle time-series curves were fitted into continuous functions employing 202 Fourier basis functions of order 3, with the smoothing parameter set to 1 × 10^−7^ (Bai-fa et al., 2019). The functional data were then decomposed into a set of principal components (PCs) through dimensionality reduction. The specific formulations are provided in Equations 1–4:

1. Calculating the covariance function v(s, t):

$$\nu \left(s,t\right)=\frac{1}{n-1}\sum_{i=1}^{n}\;\left(x_{i}\left(s\right)-\bar{x}\left(s\right)\right)\left(x_{i}\left(t\right)-\bar{x}\left(t\right)\right)$$ (1)

2. Performing an eigen decomposition of the covariance function calculates the eigenvalues λ and their corresponding eigenfunctions ξ(t) for each principal component:

$$\int \nu \left(s,t\right)\xi \left(t\right)dt=\lambda \xi \left(s\right)$$ (2)

3. The weight function is used to project the original data into the feature function space:

$$w\left(t\right)=\xi \left(t\right)\times \sqrt{\lambda }$$ (3)

4. Based on the feature function and weight function, the score of the original function data on each principal component can be calculated:

$$c_{i}=\int \xi \left(t\right)x_{i}\left(t\right)dt$$ (4)

By solving the eigen-equation of the aforementioned matrix, the eigenvalues and their corresponding eigenfunctions for each principal component were computed. The number of retained principal components was determined based on the criteria of cumulative explained variance reaching 95% and each component having an eigenvalue greater than 1 (Ryan et al., 2006). To facilitate the interpretation of the principal components, the varimax rotation method was applied, and curve registration was employed to eliminate phase variability (Ramsay and Li, 1998; Jolliffe and Cadima, 2016). Functional data analysis produced characteristic curves representing kinematic variance. In the corresponding figures, the black solid line represents the mean joint angle trajectory, while the red and blue curves depict the mean plus and minus an appropriate multiple of the principal component (PC), respectively. The envelope formed between the red and blue curves illustrates both the location and magnitude of kinematic variance (Bai-fa et al., 2019). Positive individual PC scores indicate joint angle curves approximating the red curve, whereas negative scores shift the trajectory toward the blue curve.

### 2.7 Statistical analysis

Statistical analyses were performed using Excel, MATLAB, and SPSS (version 26.0). The normality of PC scores before and after fatigue was assessed using the Shapiro-Wilk test. For data conforming to a normal distribution, paired-sample t-tests were employed, with results presented as mean ± standard deviation ( $\bar{x}$ ± s). Effect sizes were calculated using Cohen’s d, interpreted as follows: 0.2–0.5 represents a small effect, 0.5–0.8 a medium effect, and values greater than 0.8 indicate a large effect. The significance level was set at p < 0.05. For data not meeting the assumption of normality, the non-parametric Wilcoxon signed-rank test was used. Data visualization was conducted using Origin 2021.

## 3 Results

### 3.1 Functional data analysis of hip joint kinematics before and after fatigue

**FIGURE 4:**
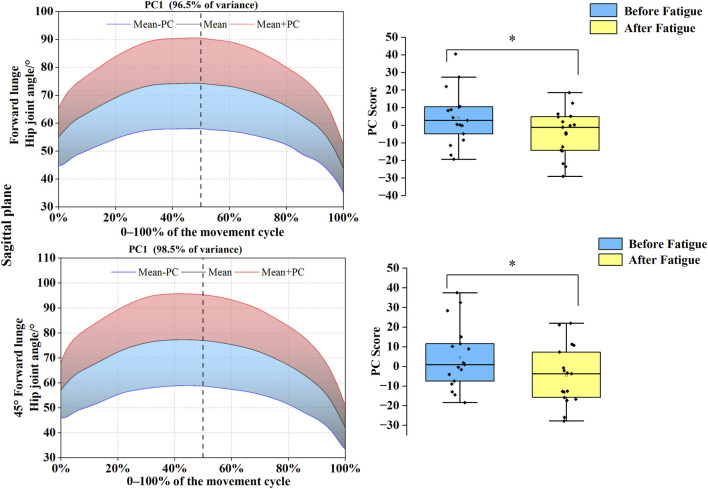
Mean hip joint angles ± weighting coefficients and principal component scores before and after fatigue. Note: Black solid line: mean joint angle trajectory. Red line: mean + a principal component (PC) multiple (Mean + PC). Blue line: mean–a PC multiple (Mean–PC). Black dashed vertical line: timing of maximum knee flexion. Blue shaded region: area of lower principal component scores (negative variation). Red shaded region: area of higher principal component scores (positive variation). PC scores are presented as mean ± standard deviation.* indicates a significant difference between conditions (p < 0.05).

**FIGURE 5:**
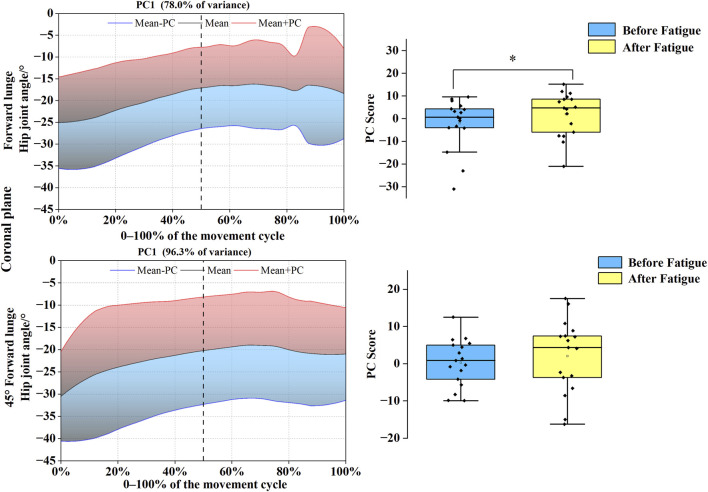
Mean hip joint angles ± weighting coefficients and principal component scores before and after fatigue (coronal plane). Note: Black solid line: mean joint angle trajectory. Red line: mean + a principal component (PC) multiple (Mean + PC). Blue line: mean–a PC multiple (Mean–PC). Black dashed vertical line: timing of maximum knee flexion. Blue shaded region: area of lower principal component scores (negative variation). Red shaded region: area of higher principal component scores (positive variation). PC scores are presented as mean ± standard deviation. * indicates a significant difference between conditions (p < 0.05).

As shown in Figures 4, 5, PC variations for the hip joint were distributed throughout the entire movement cycle in both sagittal and coronal planes. In the sagittal plane, joint angle time-series for both forward and 45° forehand lunges were reduced to a single PC, with eigenvalues of 212.92 and 274.28, accounting for 96.5% and 98.5% of the variance, respectively. Statistical analysis demonstrated significantly higher PC1 scores before fatigue compared to post-fatigue values for the forward lunge (*t* = 4.164, *p* = 0.001, Cohen’s d = 0.621) and 45° forehand lunge (*t* = 3.942, *p* = 0.001, Cohen’s d = 0.589), indicating a trajectory shift toward the blue curve after fatigue and suggesting a substantially reduced hip flexion range. In the coronal plane, both movements were similarly reduced to one PC each, with eigenvalues of 121.59 and 154.55, explaining 78.0% and 96.3% of the variance, respectively. For the forward lunge, the PC1 score was significantly lower before fatigue than after (*t* = −2.463, *p* = 0.025, Cohen’s d = 0.685), reflecting a post-fatigue shift toward the red curve and a notable decrease in the hip abduction–adduction range under fatigued conditions.

### 3.2 Functional data analysis of knee joint kinematics before and after fatigue

**FIGURE 6:**
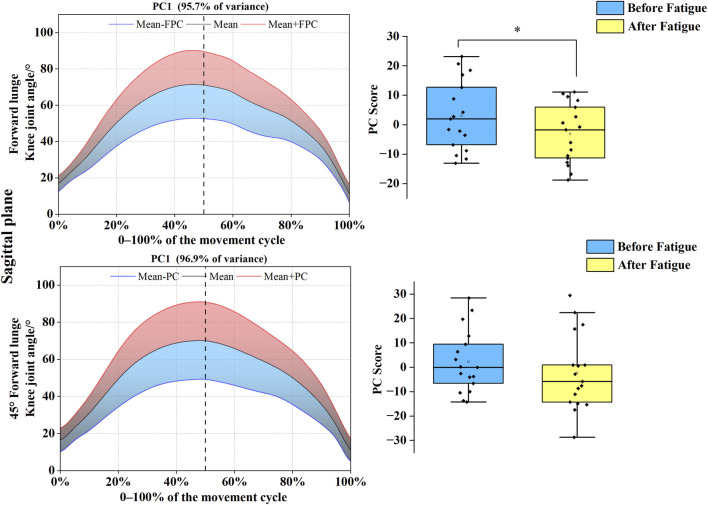
Mean knee angle ± weighting coefficient and principal component scores before and after fatigue (sagittal plane). Note: Black solid line: mean joint angle trajectory. Red line: mean + a principal component (PC) multiple (Mean + PC). Blue line: mean–a PC multiple (Mean–PC). Black dashed vertical line: timing of maximum knee flexion. Blue shaded region: area of lower principal component scores (negative variation). Red shaded region: area of higher principal component scores (positive variation). PC scores are presented as mean ± standard deviation. * indicates a significant difference between conditions (p < 0.05).

**FIGURE 7:**
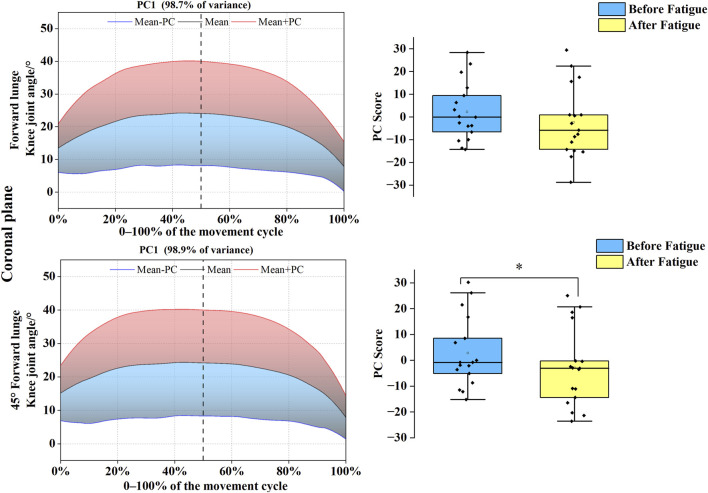
Mean knee angle ± weighting coefficient and principal component scores before and after fatigue (coronal plane). Note: Black solid line: mean joint angle trajectory. Red line: mean + a principal component (PC) multiple (Mean + PC). Blue line: mean–a PC multiple (Mean–PC). Black dashed vertical line: timing of maximum knee flexion. Blue shaded region: area of lower principal component scores (negative variation). Red shaded region: area of higher principal component scores (positive variation). PC scores are presented as mean ± standard deviation. * indicates a significant difference between conditions (p < 0.05).

As shown in Figures 6, 7, PC variations for the knee joint similarly spanned the complete movement cycle in both sagittal and coronal planes. In the sagittal plane, the joint angle time-series for both forward and 45° forehand lunges were reduced to a single PC, with eigenvalues of 193.09 and 255.93, explaining 95.7% and 96.9% of the variance, respectively. For the forward lunge, the PC1 score was significantly higher before fatigue than after (*t* = 2.509, *p* = 0.023, Cohen’s d = 0.62), indicating a post-fatigue shift toward the blue curve and a reduction in knee flexion range. In the coronal plane, both movements were likewise reduced to one PC each, with eigenvalues of 197.11 and 204.28, cumulatively accounting for 98.7% and 98.9% of the variance. Further analysis revealed that the PC1 score for the 45° forehand lunge was significantly higher before fatigue than after (*t* = 2.154, *p* = 0.031, Cohen’s d = 0.40), demonstrating a trajectory shift toward the blue curve and a significant decrease in knee abduction–adduction range under fatigue for this specific movement.

### 3.3 Functional data analysis of ankle joint kinematics before and after fatigue

**FIGURE 8:**
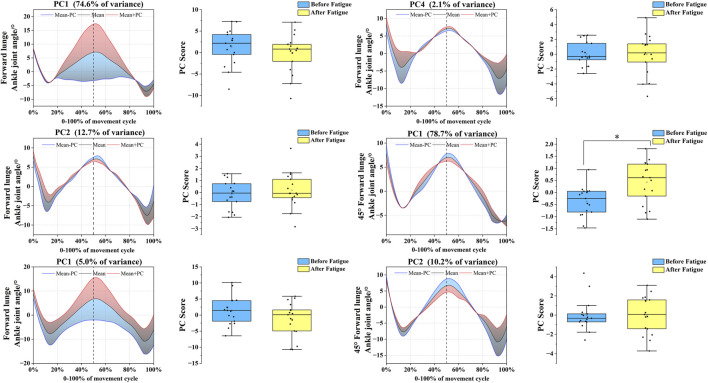
Mean ankle joint angles ± weighting coefficients and principal component scores before and after fatigue (Sagittal Plane). Note: Black solid line: mean joint angle trajectory. Red line: mean + a principal component (PC) multiple (Mean + PC). Blue line: mean–a PC multiple (Mean–PC). Black dashed vertical line: timing of maximum knee flexion. Blue shaded region: area of lower principal component scores (negative variation). Red shaded region: area of higher principal component scores (positive variation). PC scores are presented as mean ± standard deviation. * indicates a significant difference between conditions (p < 0.05).

**FIGURE 9:**
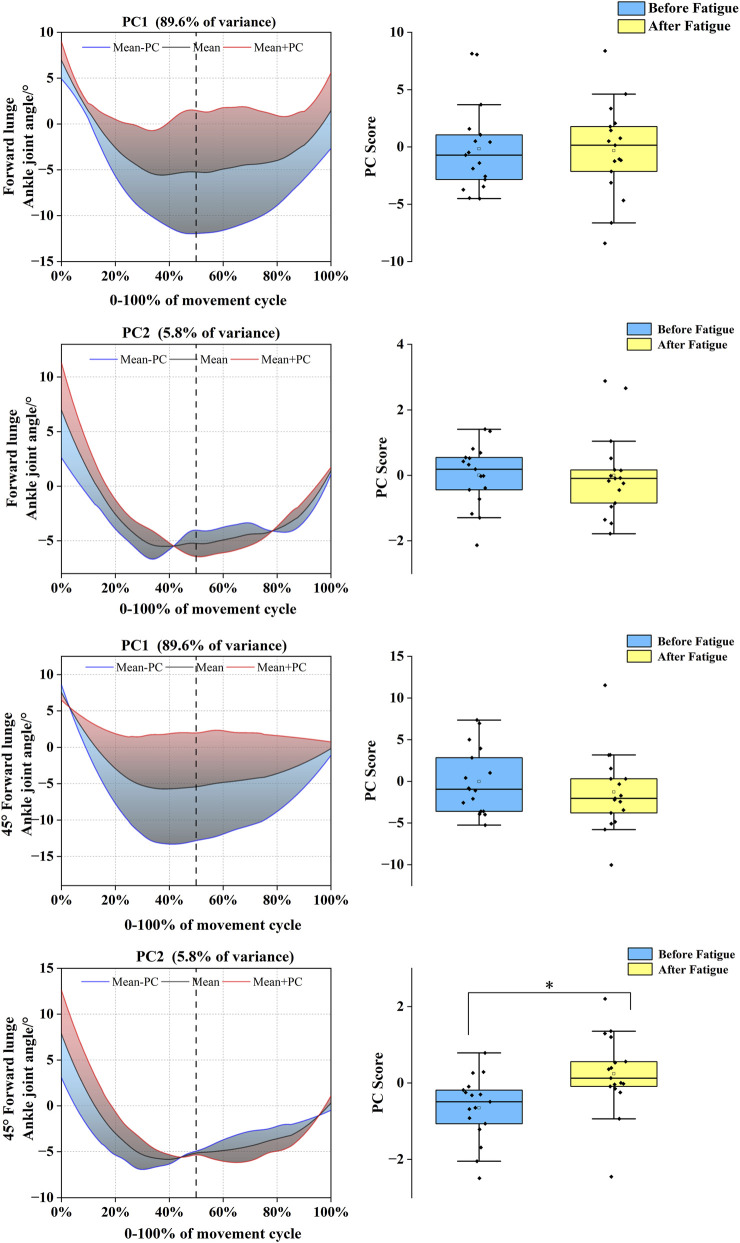
Mean ankle joint angles ± weighting coefficients and principal component scores before and after fatigue (coronal plane). Note: Black solid line: mean joint angle trajectory. Red line: mean + a principal component (PC) multiple (Mean + PC). Blue line: mean–a PC multiple (Mean–PC). Black dashed vertical line: timing of maximum knee flexion. Blue shaded region: area of lower principal component scores (negative variation). Red shaded region: area of higher principal component scores (positive variation). PC scores are presented as mean ± standard deviation. * indicates a significant difference between conditions (p < 0.05).

In the sagittal plane, the ankle joint angle time-series for the forward and 45° forehand lunges were reduced to four and two PCs, respectively. For the forward lunge, the PC eigenvalues were 28.89, 4.68, 1.64, and 1.02, cumulatively explaining 74.6%, 12.7%, 5.0%, and 2.1% of the variance; the 45° forehand lunge yielded eigenvalues of 29.14 and 3.93, explaining 78.7% and 2.1% of the variance, respectively (Figure 8). The variation associated with PC4 in the forward lunge was concentrated from the mid-late landing phase to the push-off phase. Its score was significantly lower before fatigue than after (*t* = −2.593, *p* = 0.020, Cohen’s d = 0.914), indicating a post-fatigue shift toward the red curve and an increased ankle dorsiflexion range during this phase under fatigue. In the coronal plane, both lunge movements were reduced to two PCs. The eigenvalues for the forward and 45° directions were 23.77 and 2.25, and 32.48 and 4.05, with the two PCs collectively explaining 89.6% and 5.8% of the variance, respectively (Figure 9). Further analysis revealed that PC2 for the 45° forehand lunge showed a significant difference during the early-mid landing phase, with pre-fatigue scores being lower than post-fatigue values (*t* = −2.391, *p* = 0.017, Cohen’s d = 0.795), reflecting a shift toward the red curve and a significant increase in ankle inversion–eversion amplitude after fatigue.

## 4 Discussion

This study, by introducing FDA, aimed to systematically reveal the effects of fatigue on lower-limb joint kinematics during badminton lunges from a continuous, time-varying perspective. The results demonstrated significant differences in lower-limb joint flexibility before and after fatigue. The FDA method successfully captured the dynamic evolution throughout the complete movement cycle, uncovering its phase-specific characteristics under fatigue.

At the proximal joints, FDA revealed a strategy in the hip and knee characterized by a general reduction in the range of motion. Our results showed that the first PCs for both the hip and knee joints were highly concentrated (>95% variance explained) in both sagittal and coronal planes, exhibiting a consistent cross-planar reduction in activity after fatigue. This finding corroborates the conclusions of Benjaminse et al. (2008) and Muyor and Arrabal-Campos (2016) regarding fatigue-induced movement stiffening. The underlying mechanism may involve fatigue reducing the central nervous system’s ability to recruit motor units and discharge frequency (Avela, 1998), while also impairing peripheral nerve conduction function (Filho et al., 2019), prompting the body to adopt a compensatory strategy of restricting proximal joint mobility to prioritize core stability (Yang et al., 2019). Therefore, from a neuromuscular control perspective, this stiffening can be viewed as an active adaptive adjustment. The complete time-series curves provided by FDA offer key evidence: the limitation in the range of motion persisted throughout the entire support phase, rather than being isolated to specific instants, thus corroborating from a continuous kinematics standpoint that fatigue induces a systematic, non-local reorganization of motor control strategy.

Serving as the pivotal link between proximal and distal segments, the functional behavior of the knee joint is critically dependent on proximal control (ElMelhat et al., 2022). The altered movement patterns observed at the knee in this study underscore the transmission effect of compensatory strategies along the kinetic chain. The observed reduction in knee flexion range, while potentially aiding short-term postural stability, may indicate a potential loading risk from a biomechanical perspective. This observation aligns with established literature, which suggests that a reduced knee flexion angle during landing significantly increases anterior tibial shear force—a key mechanical factor that can subsequently elevate the load on the anterior cruciate ligament (ACL) (Chappell et al., 2005; Beaulieu et al., 2023), while increasing the flexion angle serves as an effective strategy for shock absorption (Derrick et al., 2002; Elvin et al., 2007). Our study, via FDA, clearly demonstrated that the entire flexion-extension curve of the knee in the sagittal plane became “flatter” after fatigue, indicating increased dynamic stiffness and difficulty in achieving sufficient flexion during the landing phase. This functional compression of the motion range not only suggests a diminished capacity for impact absorption but also provides continuous, trajectory-level kinematic evidence consistent with the mechanism of exacerbated ACL loading. This finding appears contrary to some studies reporting increased knee flexion after fatigue (Schmitz et al., 2015; Gao et al., 2023), a discrepancy which may stem from methodological differences: discrete parameter analysis focuses on angle values at specific time points, whereas FDA, as a continuous trajectory analysis, captures movement pattern changes with different sensitivity and emphasis. In the coronal plane, the reduction in knee joint mobility can be viewed as a protective adaptation, consistent with the findings of Huang et al. (2014), who noted that athletes with knee injuries adopt a more conservative strategy, reducing coronal plane motion to decrease joint loading.

In stark contrast to the stiffening strategy of the proximal joints, the ankle joint exhibited a more complex and specific functional compensation pattern after fatigue, requiring multiple PCs for interpretation. In the sagittal plane, although the fourth functional principal component (PC4) for the forward lunge accounted for only 2.1% of the variance, its functional curve (Figure 8) revealed a distinct temporal structure characterized by an “interrupted–continued” pattern. During the mid-to-late landing phase, a positive PC4 weighting corresponded to increased ankle dorsiflexion. This trend was interrupted during the subsequent transition period and then resumed in the mid-to-late push-off phase, reflecting increased ankle plantarflexion. This phased and non-continuous modulation indicates that, under fatigue, the ankle joint does not simply increase its overall range of motion but rather undergoes a more refined, stage-specific neuromuscular reorganization. Importantly, this reorganization manifests as a strategic, phase-dependent increase in mobility. The resulting overall trend of increased ankle motion amplitude, opposite to that of the proximal joints, corroborates the theory proposed by Coventry et al. (2006) that restricting hip and knee motion during single-leg landing helps control trunk sway, while increasing the ankle motion range can more effectively buffer ground reaction forces. This mechanism is also supported by other scholars, demonstrating that increasing ankle plantarflexion and dorsiflexion range can enhance its buffering capacity, thereby reducing impact loads transmitted to the knee and ACL (Lyle et al., 2014). Therefore, our observations may reflect a movement strategy aimed at attenuating landing forces, suggesting a potential role of ankle motion in impact absorption. In the coronal plane, the ankle’s compensation pattern exhibited phase specificity. For the 45° forehand lunge, the second functional principal component (PC2) in the coronal plane explained 5.8% of the variance and showed a significant difference after fatigue. Its functional curve (Figure 9) reveals a clear, phase-specific kinematic reorganization. In the early landing phase, the joint angle at initial contact was greater after fatigue than before, indicating increased ankle inversion upon impact. Subsequently, during the mid-landing to push-off transition, the trajectory shows that the ankle progressively moves into eversion. However, in the push-off phase itself, the pattern reverses, with post-fatigue movement exhibiting reduced inversion amplitude compared to the pre-fatigue state. The increased inversion angle during the initial landing phase likely stems from fatigue-induced impairment of dynamic ankle stability. Muscle fatigue diminishes the strength and reaction speed of the evertor muscles (Li and Chiu, 2015), compromising their ability to control foot posture at ground contact and leaving the joint in a more vulnerable inverted position. This greater inversion angle increases the stress on the lateral ligaments, thereby elevating the risk of injury (Zhong et al., 2024). The reduced inversion during push-off may reflect a compensatory strategy under fatigue. This pattern resembles the diminished maximum inversion observed in patients with chronic ankle instability (CAI) (Wang et al., 2023), suggesting that fatigue can temporarily induce a similar adaptation: a purposeful limitation of inversion to lessen loading on the lateral ligaments. This could be a conservative, protective strategy that prioritizes ligament safety over movement amplitude when muscular strength and motor control are compromised.

In summary, this study systematically reveals, through Functional Data Analysis, a “proximal stiffening, distal flexibility” compensatory mode in badminton lunges under fatigue. Hip and knee joints reduce their range of motion across multiple planes, forming a top-down stiffening trend, while the ankle joint compensates distally through fine-tuned, phase-specific increases in mobility across both sagittal and frontal planes.

## 5 Conclusion

This study employed Functional Data Analysis to elucidate the “top-down” compensatory mechanism induced by fatigue within the lower-limb kinetic chain during badminton lunges. The proximal joints (hip and knee) stabilized through a consistent reduction in multi-planar motion, reflecting a neuromuscular stiffening strategy. In contrast, the distal ankle compensated via enhanced flexibility and phase-specific kinematic adjustments. Although this “proximal stiffening, distal mobilization” pattern aids in maintaining athletic performance, it does so by redistributing lower-limb biomechanical loads. This altered loading may increase the risk of injury to the ACL and the lateral ankle ligaments. By examining movement reorganization from a continuous time-varying perspective, this research advances the biomechanical understanding of badminton footwork and provides a theoretical basis for targeted training and injury prevention in fatigued athletes.

## Data Availability

The raw data supporting the conclusions of this article will be made available by the authors, without undue reservation.
